# Staplers or clips? A systematic review and meta-analysis of vessel controlling devices for renal pedicle ligation in laparoscopic live donor nephrectomy

**DOI:** 10.1097/MD.0000000000013116

**Published:** 2018-11-09

**Authors:** Yu Liu, Zhongli Huang, Yuntian Chen, Banghua Liao, Deyi Luo, Xiaoshuai Gao, Kunjie Wang, Hong Li

**Affiliations:** Department of Urology, Institute of Urology (Laboratory of Reconstructive Urology), West China Hospital, Sichuan University, Chengdu, China.

**Keywords:** clip, laparoscopic live donor nephrectomy, meta-analysis, stapler

## Abstract

**Background:**

Controlling of the renal vessels is a critical step in live donor nephrectomy (LDN). Currently, mainly 2 devices, Hem-o-Lok clips and staplers, are utilized to control vessels during LDN. Both of them have advantages and disadvantages.

**Methods:**

This systematic review and meta-analysis was aimed to compare the safety and the efficacy of the 2 devices and to identify the better one in LDN. A systematic search for related publications in the databases of PubMed, Medline, Embase, the Cochrane Library, and Web of Science through February 2018 was performed. Eight studies were selected and evaluated with the Newcastle-Ottawa Scale (NOS).

**Results:**

The meta-analysis result showed that utilization of Hem-o-Lok clips resulted in greater amount of estimated blood loss (mean differences [MD]: 40.10; 95% confidence interval [CI] 4.37–75.84) and longer time of warm ischemia (WIT) (MD: 55.61; 95% CI 36.79–74.43) than using staplers. Residual vascular length of grafts in clip group was longer than that in stapler group (MD: 2.51; 95% CI 0.78–4.24). However, the differences in primary outcomes such as device failure rate, death rate, and severe hemorrhage rate, were not significant between these 2 groups. In addition, utilization of Hem-o-Lok clips costed approximate $400 lower than staplers per patient.

**Conclusion:**

This study revealed that Hem-o-Lok clips and staplers have the similar function in LDN renal ligation, regarding the device failure rate, death rate, and severe hemorrhage rate. However, the surgeons would benefit from the clips in terms of the residue length of vessels, these outstanding features provide operation convenience and flexibility, such as right-sided donor nephrectomies, early vascular bifurcation, and rare vascular variation. In addition, the clips have potential economic advantages. In some developing countries, it would reduce the healthcare expenditure.

## 1 Introduction

Laparoscopic techniques started to be widely applied in urologic surgeries since 1990s with its first application in 1991.^[1]^ While the first live donor nephrectomy (LDN) was performed in 1995.^[2]^ Controlling of the renal pedicle is the critical step in this surgery. Safety concerns have prompted a tremendous advance in vessel ligation devices. Now various devices are available for controlling the renal pedicles, including non-absorbable polymer locking clips (Hem-o-Lok clips), titanium clips, Endo-GIA staplers, Endo-TA staplers, and so on. These devices can be roughly divided into 2 major groups, clips and staplers. In our study, utilization of Hem-o-Lok clip, Endo-GIA stapler, and Endo-TA staplers in LDN were reviewed and analyzed.

Endo-GIA stapler became the first device being used to ligate renal vessels and have been the standard ligation tool since then. It is considered to be safe and effective.^[3,4]^ However, several studies suggested the malfunctions of using Endo-GIA stapler.^[5–8]^ One of disadvantages is severe hemorrhage which required conversion from laparoscopic to open surgery or even led to the death of patient. Secondly, it provides shorter length of graft vessel for anastomosis and thirdly, the medical cost is higher.^[9]^ Therefore, surgeons have tried to secure renal pedicle with Hem-o-Lok clips since 2000 in that the clips are cheaper and can provide longer length of graft vessels.^[10]^ However, it is also associated with severe bleeding and more severely, death of the patient^[11–14]^ due to the slippage and dislodgement. Both the manufacturer of Hem-o-Lok clip and the US Food and Drug Administration (FDA) warned that Hem-o-Lok clip is contraindicated for renal artery ligation during LDN after 2006.^[11,14,15]^

Hem-o-Lok clips and staplers both showed advantages and disadvantages. To our knowledge, no studies evaluating the safety of vessel ligation devices during LDN have been published so far. This systematic review and meta-analysis study was aimed to identify the better device for vessel ligation during LDN and to provide the guidelines for clinical practice.

## 2 Methods

### 2.1 Data sources and literature search strategy

We conducted this meta-analysis in accordance with the Preferred Reporting Items for Systematic Reviews and Meta-Analyses (PRISMA) statement. Two reviewers performed a comprehensive literature search in the following databases: PubMed, Embase, and the Cochrane Library database. The last search was updated on February 2018. The publication language was restricted to English. Key words used were [“nephrectomy” AND (“Stapler” OR “stapling” OR “GIA” OR “TA” OR “gastrointestinal anastomosis” OR “Transfixion”) AND (“Clip” OR “clipping” OR “Hem-o-Lok” OR “Non-Transfixion”)]. The resulted literatures were further screened to exclude duplications followed by content screening which was achieved by title and abstract reading. After eligible studies were picked out by reading the title and abstract, a manual searching for more eligible publications were performed among the references of the literatures after content screening.

### 2.2 Inclusion and exclusion criteria

The inclusion criteria for this study were as the following: studies comparing staplers and clips used in LDN; studies provided main outcome data evaluating the safety and reliability of the devices. If ≥2 identified studies investigated the same data source or population, the largest or the most recent study was selected.

The exclusion criteria for this study were as follows: descriptive studies without comparison between clips and staplers; review articles, meta-analysis, case reports, or conference abstracts; studies based on animal or in vitro assay; studies about open surgery; duplicated studies and repeated analyses.

### 2.3 Data extraction and quality assessment

The following information was extracted from each selected study independently by 2 independent reviewers (LY and HZL). Basic data included first author, purpose of surgery, surgical approach, number of patients and type of devices. Primary outcomes were device failure rate, death rate, severe hemorrhage rate, and the cost of devices. Secondary outcomes were estimated blood loss, transfusion rate, rate of open surgery conversion, reoperation rate, residual vessel length, operation duration and warm ischemia time (WIT). If a study provided both univariate and multivariate analysis results, the multivariate analysis would be selected to achieve higher accuracy. Any discrepancies were resolved by discussion to reach a consensus. Quality of selected studies was assessed by 2 independent reviewers (LY and HZL). All the studies included were retrospective cohort studies. A study with a score ≥6 was considered high-quality study after the selected publication were evaluated with the Newcastle-Ottawa Scale (NOS).^[16]^

### 2.4 Data synthesis and statistical analysis

Data were extracted and analyzed by 2 independent researchers (LY and CYT) using Review manager 5.3 (The Cochrane Collaboration, Oxford, UK). Dichotomous variables were analyzed using risk ratio (RR), while continuous variables were analyzed using the mean differences (MD) and the corresponding 95% CIs (confidence intervals). A *P* value <.05 was considered statistically significant. Heterogeneity was quantified using the *I*^2^. A random effects model and a fixed effects model were applied for *I*^2^ > 50% and *I*^2^ < 50%, respectively. If *I*^2^ was greater than 50%, sensitivity analysis would be performed to identify the origin of heterogeneity.

### 2.5 Sensitivity analysis

To assess whether an individual study had an impact on the result, sensitivity analysis was performed for all included individual studies using the random-effects model. We examined the effects of study with the heaviest weighting by removing it and observing the change in *I*^2^ of several outcomes.

## 3 Results

### 3.1 Study selection

**Figure 1:**
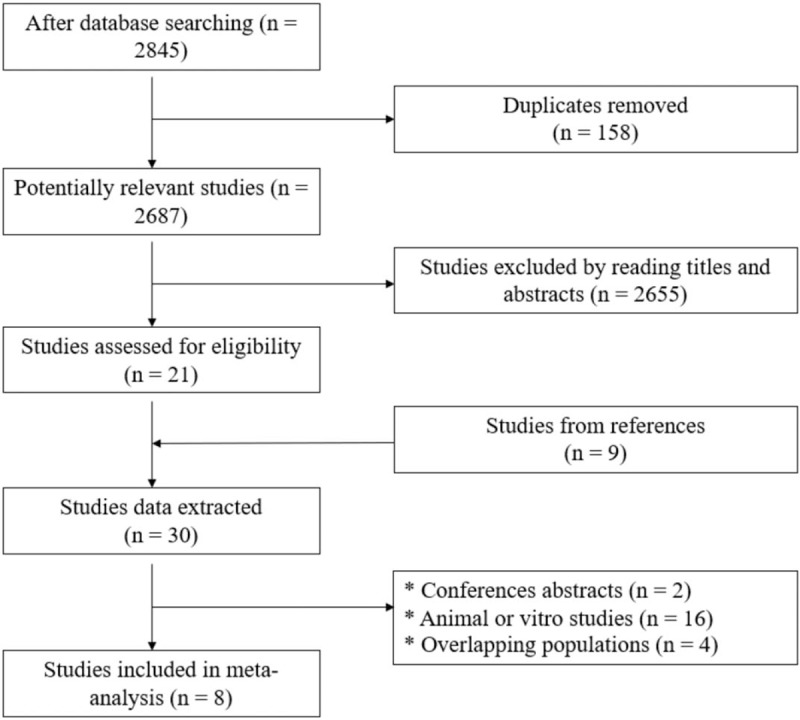
Diagram showing literature searching process.

A total of 2845 reports were found from the database search by using the described searching strategy (Fig. 1). One hundred fifty-eight duplicated articles were excluded by using Endnote X7 (Thomson Corporation, Canada). After title and abstract screening, 21 relevant studies were identified. In addition, 9 relevant studies were extracted from the references of these 21 studies, resulting in 30 publications in total. These 30 studies were read thoroughly and carefully to extract the related information. During the process, 22 articles were excluded based on the inclusion and exclusion criteria. Thus, 8 studies were finally eligible for the meta-analysis.

### 3.2 Study characteristics and quality assessment

All the selected studies were retrospective cohort studies. Regarding the surgical approaches, LDN in 5 studies were performed trans-peritoneally and 1 of them was used retro-peritoneally. The total number of included patients was 32,145 (clip group, 13,833; stapler group, 18,312) with one of the sample size was extremely large.^[15]^ Usage of Hem-o-Lok clips were found in all 8 studies. For stapler usage, 4 out 8 studies used Endo-GIA stapler, other studies used TA stapler. Three studies^[9,17,18]^ performed group comparison based on demographic characteristics and results showed no statistical difference. Besides bleeding, other existed complications included infection, ileus, and bowel injury (Table 1).^[17,22]^ All studies were scored in accordance with the NOS and the scores ranged from 7 to 9 (Table 2).

**Table 1:**
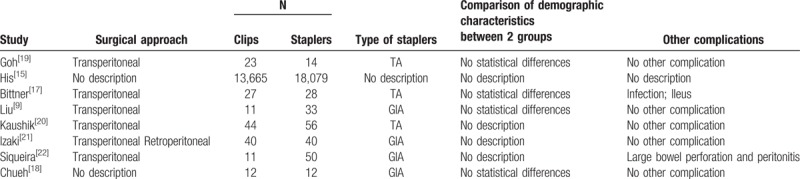
Characteristics of the reviewed studies.

**Table 2:**
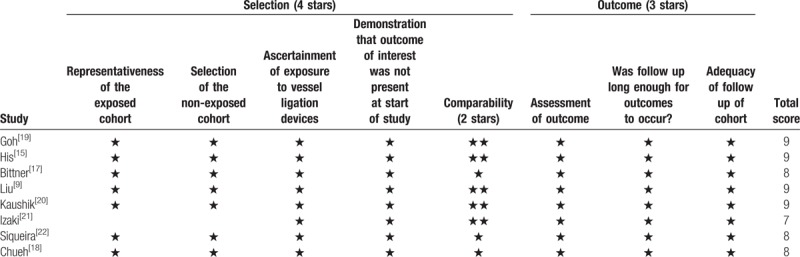
Newcastle-Ottawa Scale score of the reviewed studies.

### 3.3 Primary outcomes

**Figure 2:**
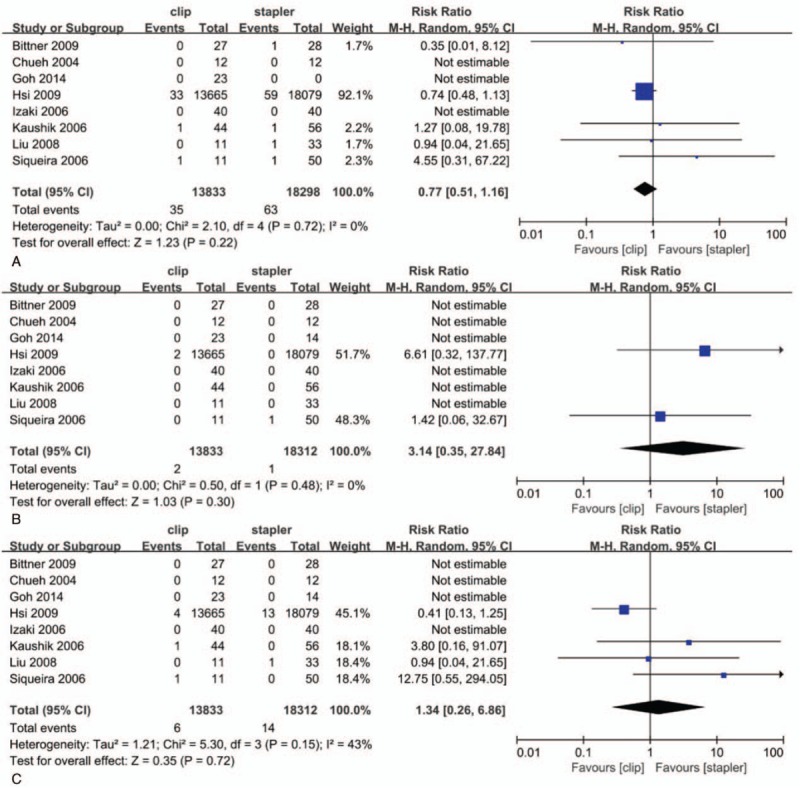
Device failure rate (A), death rate (B), and severe hemorrhage rate (C) in clip group and stapler group. Dichotomous variables were analyzed using risk ratio (RR), while continuous variables were analyzed using the mean differences (MD). 95% CIs was also calculated. A value of *P* < .05 was considered statistically significant. CI = confidence interval.

There was no significant difference between the Hem-o-Lok clips and staplers groups regarding device failure rate (risk ratio [RR]: 0.77; 95% CI: 0.51–1.16; *P* = .22), death rate (RR: 3.14; 95% CI: 0.35–27.84; *P* = .30), and severe hemorrhage rate (RR: 1.34; 95% CI: 0.26–6.86; *P* = .72) (Fig. 2). The average cost of Hem-o-Lok clips was 40 dollars, about 400 dollars (200–1440) lower than that of staplers per patient.^[20–22]^

### 3.4 Secondary outcomes

**Figure 3:**
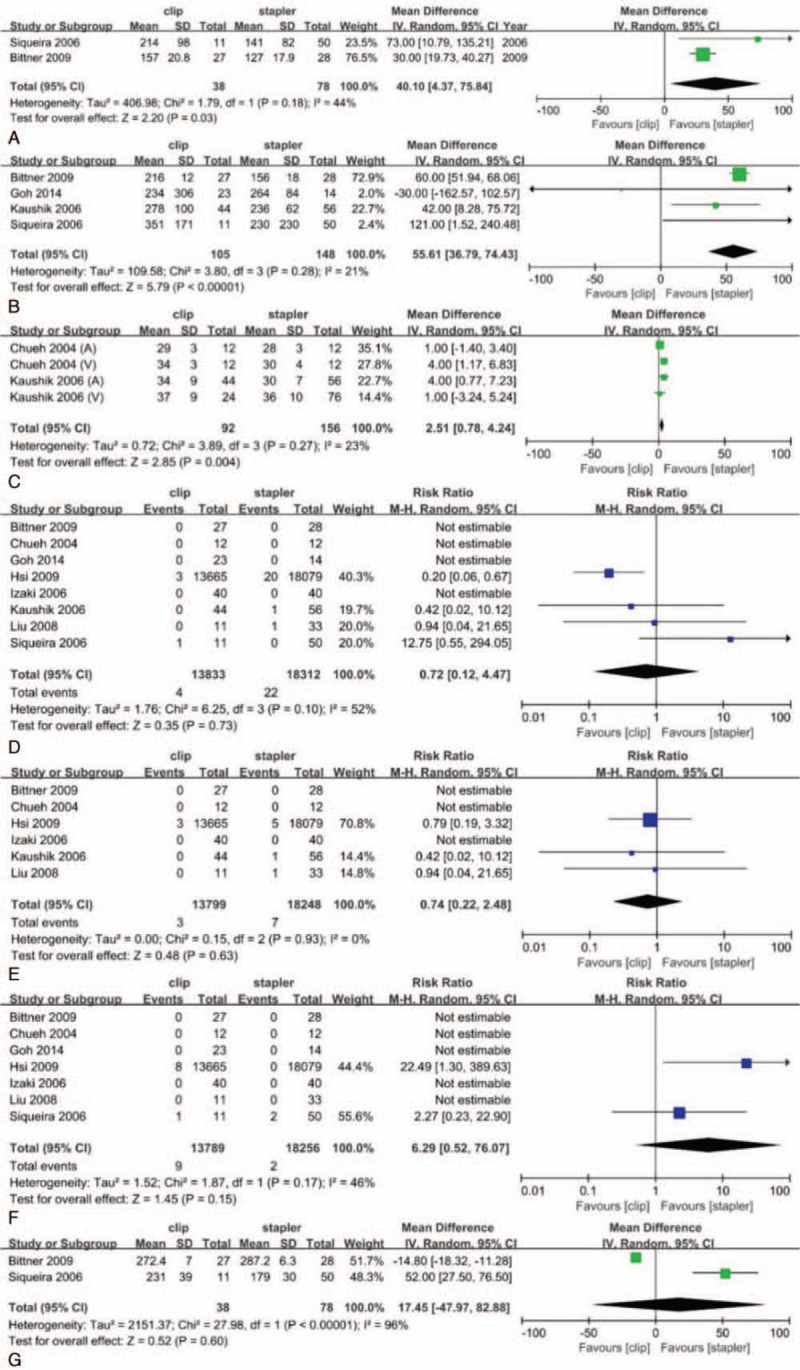
Estimated amount of blood loss (A), length of warm ischemia time (WIT) (B), residual vascular length (C), conversion to open surgery rate (D), transfusion rate (E), reoperation rate (F), and length of operative time (G) in clip group and stapler group. Dichotomous variables were analyzed using risk ratio (RR), while continuous variables were analyzed using the mean differences (MD). 95% CIs was also calculated. A value of *P* < .05 was considered statistically significant. CI = confidence interval.

The Hem-o-Lok clip group had significantly greater estimated amount of blood loss (MD: 40.10; 95% CI: 4.37–75.84; *P* = .03) and longer WIT than stapler group (MD: 55.61; 95% CI: 36.79–74.43; *P* < .001). Residual vascular length in Hem-o-Lok clip group was longer than that in stapler group (MD: 2.51; 95% CI: 0.78–4.24; *P* = .004). However, the differences of conversion rate (RR 0.72, 95% CI: 0.12–4.47, *P* = .73), transfusion rate (RR 0.74, 95% CI: 0.22–2.48, *P* = .63), reoperation rate (RR 6.29; 95% CI: 0.52–76.07; *P* = .15) and operative duration (MD: 17.45 minutes; 95% CI: 47.97–82.88; *P* = .60) were not statistically significant between the 2 groups (Fig. 3). Because of the heaviest weighting of the study performed by Hsi et al,^[15]^ we excluded it and found that there was still no difference in the severe hemorrhage rate or the conversion rate. The *I*^2^ of the severe hemorrhage rate or the conversion rate dropped from 43% and 52% to 0% and 19%, respectively. This demonstrated that the heterogeneity was mainly caused by enormous differences in sample size between studies.

## 4 Discussion

By now, various devices have been used to secure vessels including intra-corporal knot-tying,^[23,24]^ bipolar vascular sealing devices,^[25,26]^ Ligasure,^[27]^ and Harmonic Scalpel^[28–30]^ during LDN. However, those devices are only recommended for ligating the tributaries of renal vessels.^[31,32]^ Staplers and clips are 2 main devices used for controlling the renal pedicle. At the beginning of staplers application, experts thought staples would safer due to transfixion of vessel wall. However, Chan et al^[5]^ reviewed 565 laparoscopic nephrectomies performed with endovascular staplers and found that device malfunction, which was defined as failure to meet its performance expectations, occurred in 10 patients (1.7%) in 2000. In addition, other studies also reported that malfunction rate of staplers ranged from 0.2% to 1.1%.^[7,8]^ Moreover, staplers shortened the length of graft vessel and increased operative cost.^[17,20,33,34]^ Even so, 30% surgeons still preferred to use Endo-GIA staplers.^[11]^ Later on, clips were applied during the operation which theoretically compensated for the deficiency of staplers. However, several reports showing the death incidence^[15,19]^ associated with clip slippage have led to the contraindication of clips for LDN. It is unknown which device has lower rate for malfunction and complication.

Our results showed that the death rate is not significantly different between two groups. In addition, several death incidents were not related to the devices. One donor died in Hsi study due to the rupture of the artery which is proximal to clips at junction with aorta while both clips were still in place. Another donor death in Siqueira study was caused by an unrecognized large bowel perforation and peritonitis, which was not related to stapler device. Several studies have suggested that staplers might be safer than clips since staples transfix the vessel wall without slipping while titanium clips are more likely to slip^[35,36]^ owing to the nature of the clips that cannot transfix the vessel wall. Then, Hem-o-Lok clip was introduced to the field. It has teeth on jaws and can lock the mechanism at tips to reduce inadvertent dislodgment. Besides, there is a small space between the 2 jaws^[37]^ which may be more secure for those wider vessels. These improvements had led to less failure of clips, which was consistent with our results that Hem-o-Lok clips and staplers were not statistically different regarding to the device failure rate and the death rate. Notably, a potential malposition or malfunction of clips could be further avoided and controlled because the vessels are generally skeletonized. When using staplers, vessels isolation is somehow less refined, leading to a more difficult control in case of malfunction. In addition, stapling close to the aorta is theoretically riskier in case of uneventful malfunctioning since it is a single shot.

There is dramatic difference between the 2 types of devices in terms of medical cost. Our results demonstrated that clips were much cheaper than staplers. Using clips would save an average of 400 dollars for each patient which would lead to greater benefits for patients in developing countries.^[38]^ However, it should be pointed out that we didn’t make forest plot analysis for medical cost due to the lack of standard deviation data from original studies. In order to support our conclusion, we reviewed many studies and found their results were consistent with ours.^[10,20–22,33,39–41]^

Sufficient vessel length is important for renal transplantation as well. Meta-analysis result showed that Hem-o-Lok clips could provide longer length of vessels. Endo-GIA stapler ligates and transects vessels simultaneously,^[34]^ leaving 3 rows of staggered staples on each side. The trimming step after the stapling would subsequently lead to the loss of vascular length. Surgeons have tried Endo-TA stapler to make up this disadvantage of staplers. Endo-TA stapler shares the same mechanism with Endo-GIA stapler with the difference that only place 3 staple rows on the donor side.^[34]^ Sundaram et al^[32]^ found Endo-TA stapler and Hem-o-Lok clips provided longer vessel length compared with Endo-GIA stapler. Although Meng et al^[34]^ reported that residual vessels of Endo-GIA stapler were adequate for the subsequent anastomosis and the mean creatinine level was 1.6 mg/dL 45 weeks after surgery, longer vessels may still play a critical role in certain situations including right-sided donor nephrectomies, early vascular bifurcation, and rare vascular variation. If 2 renal arteries or veins occurred during the operation, the shorter length of vessels resulted from staplers would make anastomosis more complex and prolong the revascularization time and subsequently affect the graft function,^[42]^ sometimes even resulting in graft loss. Therefore, in terms of health care expenditure and the operation convenience, clips obtained more significant advantage than staplers in LDN.

During renal transplantation, WIT is important and affects the graft function. The reason why clips increased WIT is that 2 more clips were applied to control vessels and it took more time for scrub nurse to reload clips.^[33]^ However, this problem can be resolved by using 2 clip applicators.^[18,33]^ In addition, Van and Simforoosh found slightly longer WIT (no longer than 14 minutes) had no significant effect on graft function regarding the serum creatinine levels postoperatively. This concept also got support from several other studies.^[9,17,22,34,42–44]^

Some surgeons prefer to use stapler owing to the allowance of staplers to *en bloc* ligation of renal hilum even with the theoretical risks of arteriovenous fistula (AVF)^[45]^ which is associated with flank pain, cardiomegaly, and cardiac failure with high output. However, several retrospective studies^[46,47]^ had confirmed that *en bloc* ligation was safe with no AVF postoperatively. Hemal and Mishra^[48]^ preferred using Endo-GIA stapler when resecting pyonephrotic nonfunctioning kidneys to avoid high risk of adhesion, inadequate space, and inadvertent injury to surrounding tissue.

Some surgeons thought higher blood pressure might contribute to the dislodgement of clips. The leak-point pressure in cases of clips usage, ranging from 300 to 1800 mmHg, was much higher than that of staplers’. Several in vitro studies^[30,41,45,49,50]^ showed that Hem-o-Lok clips and titanium clips did not slip or lead to leakage from the end of vessel cuff under the physiological pressures. In contrast, Joseph et al^[51]^ found 4 of the 8 vascular staple lines leaked when the patients’ pressure reached 273 mmHg (237–322 mmHg) which was higher than the upper limit of normal physiological range of blood pressure. It indicated that clips may be safer than staplers for patients with supra-physiologic pressures. More in vivo and clinical studies are needed to verify this hypothesis since sometimes in vitro studies are not necessarily reflect in vivo situation.

In addition, Hem-o-Lok clips do not interfere magnetic resonance imaging (MRI) and computed tomography (CT), while metal clips are contraindicated for MRI and can lead to artifacts in CT image.

To improve the quality of our analysis and solidify the conclusion, more studies should be done. First, more data source for meta-analysis are needed. In the current study, data were extracted from only 8 studies and data about several parameters, such as death rate, estimated amount of blood loss, reoperation rate, length of operative time were extracted from only 2 out of the 8 studies. Second, all 8 studies included were retrospective studies, which may comprise some reporting bias. Third, though devices were assigned to 2 groups, staplers stapler (Endo-GIA or Endo-TA) and clips (Hem-o-Lok or titanium clip), the clips or staplers used are not exactly same. They were made by different manufactures with differences. This makes comparation between 2 devices difficult and significantly increasing the heterogenicity. Finally, several online surveys^[11,12,52]^ revealed most of the specialists had experienced either clip slippage or stapler malfunction during surgery.

## 5 Conclusions:

To summarize, our meta-analysis demonstrated that Hem-o-Lok clips and staplers have the similar function in LDN renal ligation, regarding the device failure rate, death rate, and severe hemorrhage rate. However, the surgeons would benefit from the clips in terms of the residue length of vessels, these outstanding features provide operation convenience and flexibility, such as right-sided donor nephrectomies, early vascular bifurcation, and rare vascular variation. In addition, the clips have potential economic advantages. In some developing countries, it would reduce the healthcare expenditure.

## Author contributions

**Conceptualization:** Yu Liu, Zhongli Huang, Banghua Liao, Kunjie Wang, Hong Li.

**Critical revision of the manuscript:** Kunjie Wang, Hong Li.

**Data acquisition:** Yu Liu, Zhongli Huang.

**Data analysis:** Yu Liu, Zhongli Huang, Yuntian Chen, Banghua Liao, Deyi Luo, Xiaoshuai Gao.

**Data curation:** Yu Liu, Zhongli Huang, Deyi Luo.

**Drafting of the manuscript:** Yu Liu.

**Formal analysis:** Yu Liu, Zhongli Huang, Xiaoshuai Gao.

**Funding acquisition:** Banghua Liao.

**Investigation:** Xiaoshuai Gao.

**Methodology:** Yu Liu, Zhongli Huang, Yuntian Chen, Banghua Liao, Xiaoshuai Gao.

**Project administration:** Yu Liu, Deyi Luo, Kunjie Wang.

**Resources:** Yu Liu, Zhongli Huang.

**Software:** Zhongli Huang, Yuntian Chen, Deyi Luo, Xiaoshuai Gao.

**Study design:** Yu Liu, Zhongli Huang, Yuntian Chen, Kunjie Wang, Hong Li.

**Supervision:** Yu Liu, Yuntian Chen, Banghua Liao, Deyi Luo, Kunjie Wang, Hong Li.

**Validation:** Zhongli Huang, Yuntian Chen, Banghua Liao, Deyi Luo, Kunjie Wang, Hong Li.

**Visualization:** Deyi Luo, Hong Li.

**Writing – original draft:** Yu Liu.

**Writing – review & editing:** Banghua Liao, Deyi Luo, Kunjie Wang, Hong Li.
